# Prenatal diagnosis of auriculocondylar syndrome with a novel missense variant of *GNAI3*: a case report

**DOI:** 10.1186/s12884-021-04238-x

**Published:** 2021-11-17

**Authors:** Xiaoliang Liu, Wei Sun, Jun Wang, Guoming Chu, Rong He, Bijun Zhang, Yanyan Zhao

**Affiliations:** 1 Department of Clinical Genetics, Shengjing Hospital of China Medical University, Shenyang, China; 2 Department of Ultrasonography, Shengjing Hospital of China Medical University, Shenyang, China; 3 Department of Obstetrics and Gynecology, Shengjing Hospital of China Medical University, Shenyang, China

**Keywords:** Auriculocondylar syndrome, *GNAI3*, Craniofacial deformity, Prenatal diagnosis, Case report

## Abstract

**Background:**

Auriculocondylar syndrome (ACS) is a rare disorder characterized by micrognathia, mandibular condyle hypoplasia, and auricular abnormalities. Only 6 pathogenic variants of *GNAI3* have been identified associated with ACS so far. Here, we report a case of prenatal genetic diagnosis of ACS carrying a novel *GNAI3* variant.

**Case presentation:**

A woman with 30 weeks of gestation was referred to genetic counseling for polyhydramnios and fetal craniofacial anomaly. Severe micrognathia and mandibular hypoplasia were identified on ultrasonography. The mandibular length was 2.4 cm, which was markedly smaller than the 95th percentile. The ears were low-set with no cleft or notching between the lobe and helix. The face was round with prominent cheeks. Whole-exome sequencing identified a novel de novo missense variant of c.140G > A in the *GNAI3* gene. This mutation caused an amino acid substitution of p.Ser47Asn in the highly conserved G1 motif, which was predicted to impair the guanine nucleotide-binding function. All ACS cases with *GNAI3* mutations were literature reviewed, revealing female-dominated severe cases and right-side-prone deformities.

**Conclusion:**

Severe micrognathia and mandibular hypoplasia accompanied by polyhydramnios are prenatal indicators of ACS. We expanded the mutation spectrum of *GNAI3* and summarized clinical features to promote awareness of ACS.

## Background

Auriculocondylar syndrome (ACS, MIM#602483, #614669 and #615706) is an extremely rare monogenic condition affecting neural crest cell development within the first and second pharyngeal arches [1]. It is characterized by craniofacial malformations with a triad of core features: micrognathia, mandibular condyle hypoplasia, as well as a distinctive question mark ear (QME) with a cleft or notching between the lower helix and the lobule. Other features, including microstomia, palatal abnormalities, glossoptosis, respiratory distress, round face with prominent cheeks, facial asymmetry, hearing loss, and postauricular tags, may be manifested [2]. Marked inter- and intra-familial phenotypic variability and incomplete penetrance have been observed in ACS [3, 4]. Genetic variants underlying ACS have been identified in three genes: guanine nucleotide binding protein alpha-inhibiting activity polypeptide 3 (*GNAI3*), phospholipase C beta 4 (*PLCB4*), and endothelin 1 (*EDN1*), all of which are implicated in the EDN1-endothelin type A receptor (EDNRA) pathway [2, 5]. Gαi3, encoded by the *GNAI*3 gene, is a member of the heterotrimeric G proteins. It has five guanine nucleotide-binding sites of G1–G5 boxes in the guanosine triphosphate catalytic domain [6]. So far, only 6 mutations of *GNAI*3 have been identified in autosomal dominant type I ACS, all of which affected the conserved amino acid positions that cluster within the nucleotide binding pocket [3, 4, 7, 8].

In the present study, we report a prenatal genetic diagnosis case of ACS in the Chinese population. A novel missense variant of *GNAI3* was identified in the G1 box, which was predicted to impair the guanine nucleotide-binding function. The clinical features of all ACS cases with *GNAI3* mutations described in the literature were reviewed.

## Case presentation

**Fig. 1:**
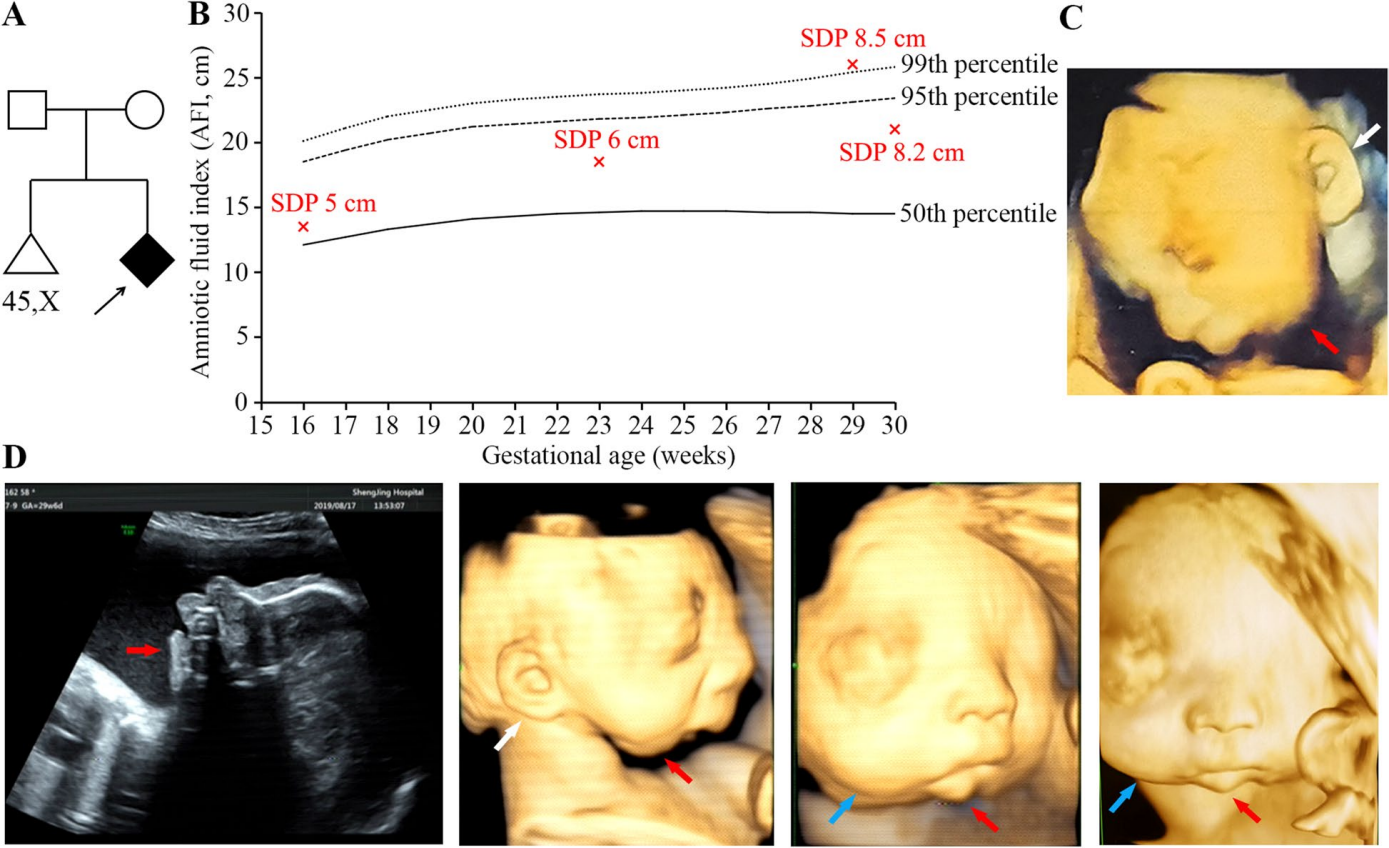
**A** Pedigree of a Chinese prenatal case with auriculocondylar syndrome (ACS). The proband, an affected fetus, is depicted as a filled diamond and indicated by a black arrow. **B** Recording of the amniotic fluid indexes (AFIs) measured by ultrasonography. The median, upper 95th, and upper 99th percentiles of AFI in normal singleton pregnancy are plotted as solid, dashed, and dotted lines, respectively (data from Moore & Cayle, [9]). The AFIs of this case are indicated by red crosses and the single deepest pockets (SDPs) are also presented accordingly. **C** Craniofacial features of the affected fetus visualized by ultrasonography at 29 weeks of gestation. **D** Craniofacial features of the affected fetus visualized by ultrasonography at 29 weeks and 6 days of gestation. The mandibular hypoplasia (severe micrognathia, retrognathia, short and vertical mandibular ramus, linear configuration of the mandibular body, and loss of mandibular angle) is indicated by white arrows; the low-set ears are indicated by white arrows; the round face is indicated by blue arrows

A 27-year-old gravida 2, para 0 Chinese woman (body weight: 62 kg, height: 162 cm) was referred to genetic counselling at 30 weeks of gestation due to polyhydramnios and fetal craniofacial deformities. She had experienced first-trimester spontaneous pregnancy loss of a 45,X embryo. She denied consanguineous marriage to her 28-year-old husband, or any exposure to teratogens. The couple showed no facial abnormalities, facial asymmetry, or micrognathia (Fig. 1A).

Routine antenatal examinations of this pregnancy were performed at her local county hospital, and maternal hyperthyroidism was diagnosed and treated with regular drugs since 10 weeks of gestation. Ultrasonography has been made at 8, 12, 16, 23 and 29 weeks of gestation. Attention was drawn by the continuously high amniotic fluid volume since 16 weeks, especially by the amniotic fluid index (AFI) of 25 cm and single deepest pocket (SDP) of 8.5 cm at 29 weeks to diagnose polyhydramnios (Fig. 1B) [9]. Micrognathia was not recognizable in the printed reports from the local hospital until 29 gestational weeks. The left ear was low-set with no cleft or notching between the helix and the lobule (Fig. 1C). The fetus was pinpointed for mandibular hypoplasia at our hospital by fetal anomaly scan at 29 weeks and 6 days of gestation (Fig. 1D), showing severe micrognathia, retrognathia, short and vertical mandibular ramus, linear configuration of the mandibular body, and loss of mandibular angle. The mandibular length was 2.4 cm, which was markedly smaller than that of normal singleton fetuses at 29 weeks of gestation (median: 4.08 cm; lower 95th percentile: 3.8 cm) [10]. The right ear was low-set and semicircular in shape, with no cleft or notching. The face was round with prominent cheeks. No facial asymmetry was observed. The SDP and AFI was 8.2 and 21 cm, respectively. The fetal stomach bubble was 2.78 cm × 0.96 cm, which was comparable to the normal gastric size of 28–30 weeks gestational age (2.23 ± 1.54 cm × 1.28 ± 1.44 cm) [11]. No other structural anomalies were found in the fetus.

**Fig. 2:**
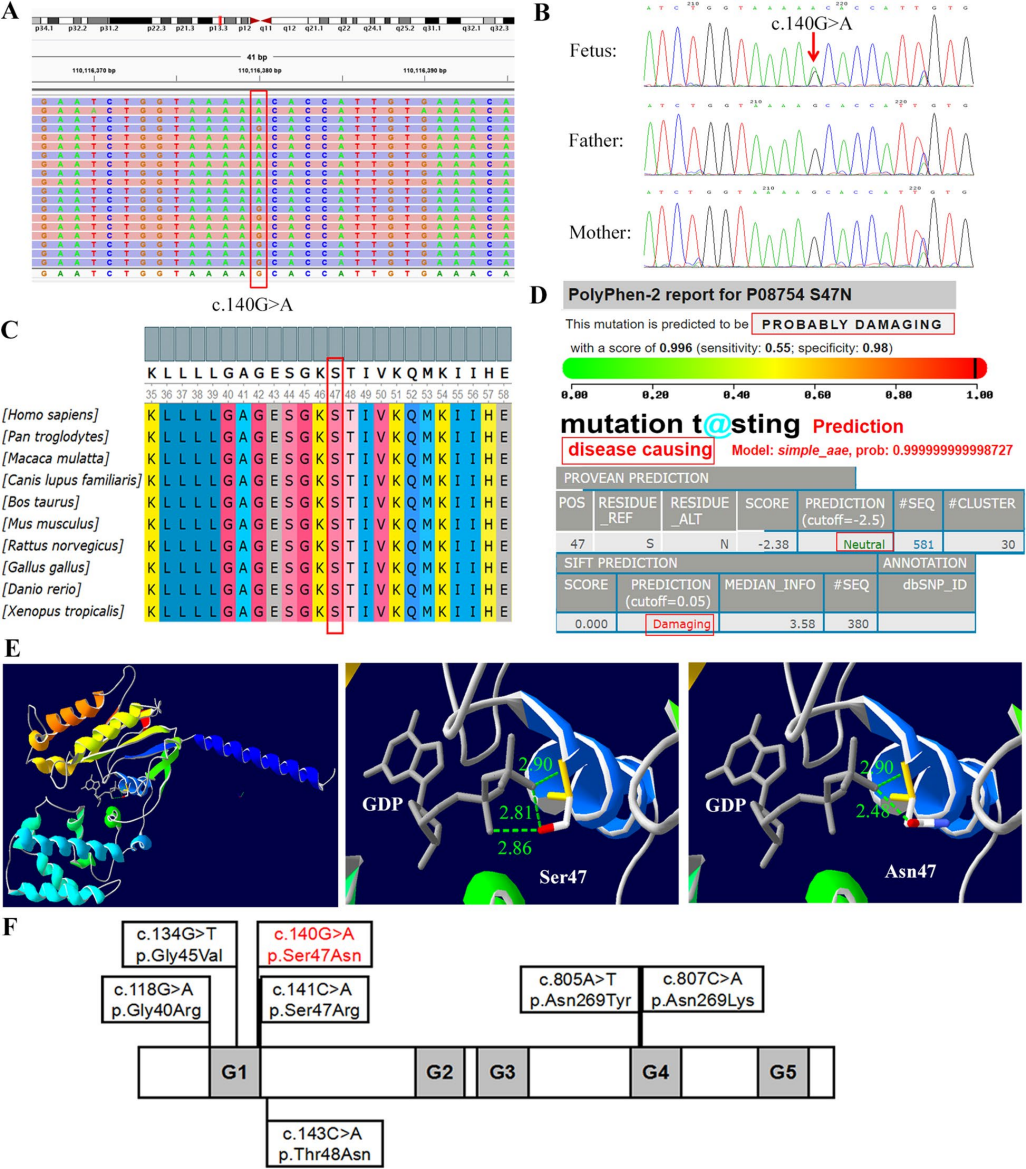
**A** Whole exome sequencing of the *GNAI3* heterozygous c.140G > A variant (indicated in a red box). **B** Sanger sequencing of the *GNAI3* heterozygous c.140G > A variant (indicated by a red arrow). **C** Evaluation of the amino acid conservation by Ugene. Each residue in alignment is assigned a color according to the ClustalX color scheme. The Ser47 of Gαi3 (indicated in a red box) is highly conserved accross various species. **D** In silico prediction of the *GNAI3* c.140G > A variant by PolyPhen, MutationTaster, PROVEAN, and SIFT. **E** The three dimensional molecular structure of Gαi3 and the guanosine diphosphate (GDP) ligand by SWISS-MODEL and Swiss-PdbViewer. The overview (left panel) and magnified views of the wild-type Ser47 (middle panel) and mutant Asn47 (right panel) are shown accordingly. The H-bonds to GDP are shown as green dashed lines, with H-bond distances (Å) shown in green numbers. **F** Schematic representation of all the identified mutations in the *GNAI3* gene (NM_0064). Five guanine nucleotide-binding sites (G1–G5) are indicated by gray boxes. The novel missense mutation in the present report is shown in red

Amniocentesis was performed to seek for potential genetic defects in the fetus. Chromosomal aneuploidy and rearrangement were excluded by low-coverage massively parallel CNV-sequencing, which yielded normal results (data not shown). A heterozygous variant of c.140G > A (NM_0064) was found in exon 2 of the *GNAI3* gene by whole exome sequencing (WES, Fig. 2A). Further Sanger sequencing confirmed the variant to be de novo that it did not occur in the parents (Fig. 2B). It was a missense mutation that caused amino acid substitution of p.Ser47Asn. The Ser47 of Gαi3 is highly conserved across various species (assayed by Ugene, Fig. 2C). Four in silico tools of PolyPhen, MutationTaster, PROVEAN, and SIFT were used to predict the harmfulness of this missense variant, showing all deleterious except for “Neutral” by PROVEAN (Fig. 2D). The Ser47 resided in the last amino acid of the G1 box. The 3D molecular model was built using SWISS-MODEL, and the H-bonds toward guanosine diphosphate (GDP) were calculated by Swiss-PdbViewer. As shown in Fig. 2E, the substitution of Ser47 to Asn47 changed the sidechain direction and disrupted one of the three H-bonds to GDP, which might interfere with the binding and downstream signaling of the G protein. The variant was novel that has been neither reported in literature nor found in the public or inhouse database. This represents the seventh variant to the *GNAI3* mutation spectrum in ACS, which was schematically depicted in Fig. 2F.

By reviewing literature, 19 individuals have been identified carrying 7 distinct missense variants from 8 cases, among which 4 were familial and 4 were sporadic (Table 1) [3, 4, 7, 8, 12–15]. Two individuals with *GNAI3* mutations (p.Gly40Arg and p.Asn269Tyr) were normal and the penetrance of the *GNAI3*-underlying ACS was 89.4% (17/19). Being autosomal dominant, the number of female and male individuals carrying mutations were mostly equal (10:9). However, the ratio of female to male probands, who were usually the most severe in the family, was 6:2. Moreover, nearly all the individuals with tracheostomy (7/8, 87.5%) and gastrostomy (7/7, 100%) were females, indicating female-dominated severe cases. The overall expressivity of clinical characters was summarized, showing common features of micrognathia (78.95%), auricular malformation (68.42%), microstomia (66.67%), prominent cheeks (63.16%), mandibular hypoplasia (57.89%), and round faces (57.89%). Four patients showed asymmetric mandibular and auricular malformations that were either more severe on the right or right ear affected only, indicating right-side-prone deformities. The prenatal findings of two patients were retrospectively described in brief. Together with ours, all the three showed polyhydramnios (100%), and two were noticed for micrognathia (66.67%) in the ultrasonography.

**Table 1:** Clinical features of reported auriculocondylar syndrome (ACS) cases with *GNAI3* (NM_0064) mutations

	c.118G > C p.Gly40Arg		c.134G > T p.Gly45Val	c.140G > A p.Ser47Asn	c.141C > A p.Ser47Arg	c.143C > A p.The48Asn	c.805A > T p.Asn269Tyr	c.807C > A p.Asn269Lys	Summary
Publications	Rieder 2012 (S008)	Erlich 2000; Rieder 2012 (S011)	Propst 2013; Romanelli Tavares 2015 (SP1)	This study	Gordon 2013 (Case 7)	Tavares 2015 (SP2)	Guion-Almeida 2002 (Patient 1); Masotti 2008 (F2); Romanelli Tavares 2015 (ACS1)	Yanagi 2021	
Familial/sporadic	Familial	Familial	Sporadic	Sporadic	Familial	Sporadic	Familial	Sporadic	1:1
Individuals with mutation	2 (1 F; 1 M)	2 (2 F, 0 M)	1 (M)	1 (F)	2 (1 F, 1 M)	1 (M)	9 (4 F, 5 M)	1 (F)	19 (10 F, 9 M)
Penetrant patients	1/2	2/2	1/1	1/1	2/2	1/1	8/9	1/1	17/19 (89.47%)
Proband sex	F	F	M	F	F	M	F	F	8 (6 F, 2 M)
**MANDIBLE**									
Micrognathia	1/2	2/2	1/1	1/1	2/2	1/1	6/9	1/1	15/19 (78.95%)
Condyle hypoplasia	1/2	2/2	1*/1	1/1	2*/2	1*/1	2/9	1/1	11/ 19 (57.89%)
**EAR**									
Auricular malformation	1/2	2/2	1*/1	0/1	1^#^/2	1^#^/1	6/9	1/1	13/19 (68.42%)
Hearing loss	NA	2/2	1/1	NA	0/2	1/1	2/9	1/1	7/16 (43.75%)
**MOUTH**									
Microstomia	1/2	2/2	1/1	NA	2/2	1/1	4/9	1/1	12/18 (66.67%)
Glossoptosis	1/2	2/2	1/1	NA	0/2	1/1	2/9	1/1	8/18 (44.44%)
Abnormal palate	NA	2/2	1^a^/1	NA	0/2	1^b^/1	1/8	0/1	5/15 (33.33%)
**FACE**									
Prominent cheeks	1/2	2/2	1/1	1/1	2/2	1/1	3/9	1/1	12/19 (63.16%)
Round faces	1/2	2/2	1/1	1/1	2/2	1/1	2/9	1/1	11/19 (57.89%)
Asymmetry	0/2	0/2	0/1	0/1	2*/2	1*/1	0/9	0/1	3/19 (15.79%)
**OTHER**									
Respiratory distress	1^&^/2	2^&^/2	1/1	NA	NA	1/1	2^&^/9	1^&^/1	8/16 (50%)
Feeding difficulties	1^&^/2	2^&^/2	1/1	NA	NA	1/1	1^&^/9	1^&^/1	7/16 (43.75%)
**PRENATAL**^**$**^									
Polyhydramnios	NA	1/1	NA	1/1	NA	NA	1/1	NA	3/3 (100%)
Micrognathia	NA	1/1	NA	1/1	NA	NA	0/1	NA	2/3 (66.67%)

Abbreviations: *M* male; *F* female; *NA* not reported

*: More severe on the right; #: right ear only; &: severe cases with tracheostomy or gastrostomy; $: evaluated only in the proband; a: high-arched palate; b: bifid uvula

The couple in the present case were informed of the genetic diagnosis of ACS, the details of this rare syndrome, and the recurrent risk in their future pregnancies. They eventually opted for pregnancy termination at the local hospital at 33 weeks and 6 days of gestation and refused any photography and post-mortem autopsy of the fetus.

## Discussion and conclusion

ACS is an extremely rare disorder that was first described in 1998, first mapped a locus to 1p21.1-q23.3 in 2008, and first identified for causative genes in 2012 [7, 15, 16]. The rarity limits our understanding of the disease, especially with regard to the prenatal features of the disorder. In the present singleton fetus, the amniotic fluid volume had been high since 16 weeks of gestation, and met the criteria of polyhydramnios at 29 weeks. Prenatal ultrasound diagnosis of micrognathia has been reported as early as first trimester by experienced examiner [17]. In the present case however, micrognathia was overlooked in the local hospital from the nonstandard sections in printed reports at early gestational weeks. To improve detection of micrognathia, it is important to take the mid-sagittal plane of fetal facial profile. Objective assessments are required for less severe fetal micrognathia, including direct measurement of mandible length or indirect evaluation from angles of facial profiles such as inferior facial angle (IFA), facial maxillary angle (FMA), frontal nasomental angle (FNMA), and fetal profile line (FPL) [18]. Micrognathia and mandibular hypoplasia were confirmed in our hospital in the fetal anomaly scan from the homeotic transformed maxillary-like structures including short and vertical mandibular ramus, linear configuration of the mandibular body, and loss of mandibular angle. Two previous reports have mentioned about the prenatal ultrasonic findings of ACS, showing polyhydramnios in both and micrognathia in one fetus whose affected mother aroused early attention at 19 weeks of gestation [12, 14]. The polyhydramnios was secondary to inefficient swallowing of amniotic fluid, which arose from temporomandibular joint ankylosis, microstomia, glossoptosis or tongue/soft-tissue abnormalities. The fetal stomach bubble was not small in our fetus with dysphagia. Prenatal auricle defects have been reported together with multiple deformities in other syndromes of the first and second pharyngeal arches [19, 20]. In the two reports of ACS patients (QME positive) with brief prenatal descriptions, ear abnormalities were not mentioned [12, 14]. The ears of our fetus were low-set, but unexpectedly not in accord with the characteristic QME. The face was round and symmetrical, with prominent cheeks which might be easily overlooked. Collectively, severe hypoplastic mandible and the accompanying polyhydramnios could be considered as prenatal indicators of ACS.

ACS is genetically heterogeneous with pathogenic variants identified in the *GNAI3*, *PLCB4*, and *EDN1* genes. They encode signaling molecules in the EDN1-EDNRA pathway, which is important for patterning the mandibular portion of the first pharyngeal arch [2, 5]. Gαi3 forms the heterotrimeric G protein with Gβγ to transduct the EDN1-EDNRA signal to PLC, which induces the expression of transcription factors DLX5 and DLX6 required for the differentiation of neural crest cells [2]. All the currently known *GNAI3* mutations were autosomal-dominant and missense, and structurally resided in the G1 and G4 boxes involved in binding guanine nucleotides [3, 4, 7, 8]. The fetus in this report carried a novel de novo p.Ser47Asn mutation in the conserved G1 box. A dominant negative effect of ACS-associated mutant Gαi3 has been proposed in that the mutant proteins were capable of EDNRA coupling but defective in guanosine binding using an in vitro *Xenopus* model [6]. The substitution of Ser47 into Asn47 was predicted to be deleterious as it disrupted one of the three H-bonds toward GDP, which might thereafter disrupt the EDN1-EDNRA signaling pathway.

A wide range of phenotypic variability and incomplete penetrance have been observed in ACS [3, 4]. We summarized the clinical features of all identified ACS cases with *GNAI3* gene mutations including ours, showing a penetrance of 89.47%. Overall, the most common features included micrognathia, auricular malformation, microstomia, prominent cheeks, mandibular hypoplasia, and round faces. Further, there were two interesting observations in *GNAI3*-related ACS: severe cases were female-dominated and deformities were right-side-prone. More cases are needed to clarify these trends, and more functional experiments are needed to elucidate possible explanations. QME is the characteristic auricular malformation in ACS, with severity varying from complete cleft to a mild notching between the helix and lobule. Our fetus with the p.Ser47Asn mutation showed normal ears. A previous familial case with p.Ser47Arg mutation showed completely normal ears in the father and a only right ear constriction in the daughter [4]. In addition, a boy with p.The48Asn showed only right-side QME [3]. These cases might infer a mild ear phenotype by *GNAI3* mutations at the G1 boundary of Ser47 and The48. In comparison to the mandibular phenotype, our fetus with p.Ser47Asn mutation was more severe than the patients with p.Ser47Arg mutation [4]; and the patient carrying p.Asn269Lys mutation was also more severe than the patients carrying p.Asn269Tyr mutation [8, 14]. A possible explanation might be that the substitution to different amino acids at the same position destruct the GDP binding at different degrees: Arg47 holds three H-bonds to GDP compared to two by Asn47 (analyzed by Swiss-PdbViewer, data not shown); and the hydrophobic interaction might be easily disrupted by Lys269 than Tyr269 [8].

In conclusion, severe micrognathia and mandibular hypoplasia accompanied by polyhydramnios are prenatal indicators of ACS. A novel missense variant of *GNAI3* was identified in the G1 box to impair the guanine nucleotide-binding function. The phenotypic parameters of all previous ACS cases with *GNAI3* gene mutations were summarized to improve awareness of this rare disorder.

## Data Availability

The datasets obtained and/or analyzed during the current study are available from the corresponding author on reasonable request.

## Abbreviations

ACS: Auriculocondylar syndrome
QME: Question mark ear
GNAI3: Guanine nucleotide binding protein alpha-inhibiting activity polypeptide 3
PLCB4: Phospholipase C beta 4
EDN1: Endothelin 1
EDNRA: Endothelin type A receptor
AFI: Amniotic fluid index
SDP: Single deepest pocket
WES: Whole exome sequencing
GDP: Guanosine diphosphate
